# Supplementary material for: Activation of melanocortin-1 receptor signaling in melanoma cells impairs T cell infiltration to dampen antitumor immunity
Source: Nat Commun. 2023 Sep 15;14:5740. doi: 10.1038/s41467-023-41101-3 (PMC10504282; doi:10.1038/s41467-023-41101-3)

Cui et al: Activation of melanocortin-1 receptor signaling in melanoma cells impairs T cell infiltration to dampen antitumor immunity

Supplementary information

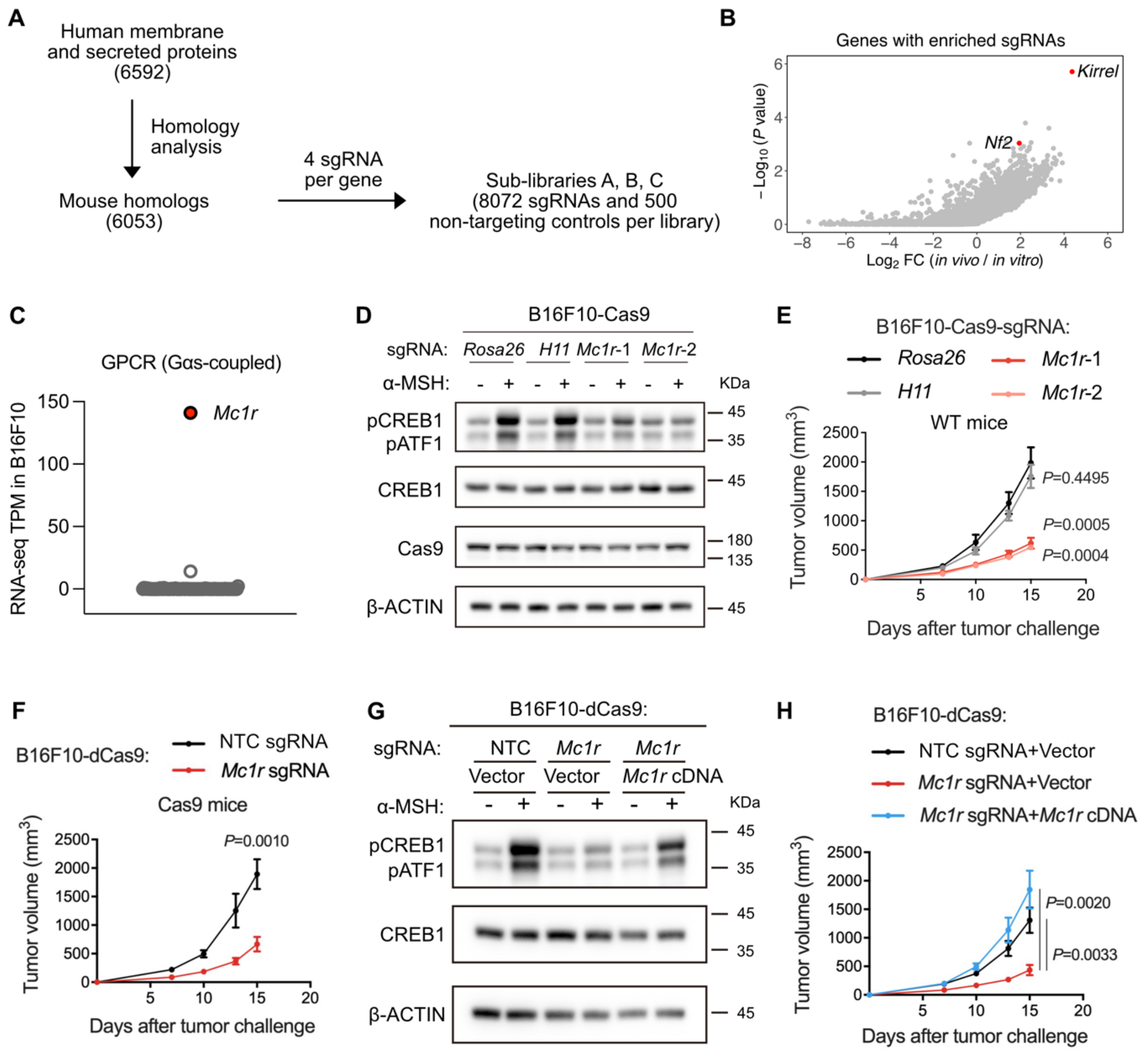

Figure S1. CRISPR screening and *MC1R* validation.

(A) Design of custom sgRNA libraries targeting genes encoding membrane or secreted proteins conserved between human and mouse.

(B) Scatterplot depicting genes with significantly enriched sgRNAs in B16F10-Cas9 tumors (n=8, 6, 7 independent tumors respectively for sub-libraries A, B, C) versus cultured B16F10-Cas9 cells. Genes were plotted based on mean $\log_2$ fold change ($\log_2$FC) of sgRNA counts in B16F10-Cas9

tumors compared to cultured B16F10-Cas9 cells and *P* values computed by MAGeCK. Source data are provided as a Source Data file.

(C) Expression levels (tag per million RNA-seq reads) of 86 Gαs-coupled GPCR (defined by GPCRdb) in B16F10. Source data are provided as a Source Data file.

(D) Western blotting of the indicated proteins in B16F10-Cas9 cells transduced with indicated sgRNAs. Cells were untreated or treated with 1 μM α-MSH for 1 hour. A representative result was shown from two independent experiments. Uncropped western blot images are provided in Figure S11.

(E) Tumor volume of wild-type mice transplanted with B16F10-Cas9 tumors expressing indicated sgRNA. Data are the mean ± s.e.m. (n=10 for *Rosa26* sgRNA; n=9 for *H11* sgRNA, n=9 for *Mc1r* sgRNA-1, n=10 for *Mc1r* sgRNA-2). Welch's t-tests (two-tailed) were used to determine the statistical significance of the differences in tumor volume. Source data are provided as a Source Data file.

(F) Tumor volume of Cas9 transgenic mice transplanted with B16F10-dCas9 tumors expressing indicated sgRNA. Data are the mean ± s.e.m. with n=10 animals per group. Welch's t-tests (two-tailed) were used to determine the statistical significance of the differences in tumor volume. Source data are provided as a Source Data file.

(G) Western blotting of the indicated proteins in B16F10-Cas9 cells transduced with indicated sgRNAs and cDNA. Cells were untreated or treated with 1 μM α-MSH for 1 hour. A representative result was shown from two independent experiments. Uncropped western blot images are provided in Figure S11.

(H) Tumor volume of wild-type mice transplanted with B16F10-Cas9 cells transduced with indicated sgRNAs and cDNA. Data are the mean ± s.e.m. with n=10 animals per group. Welch's t-tests (two-tailed) were used to determine the statistical significance of the differences in tumor volume. Source data are provided as a Source Data file.

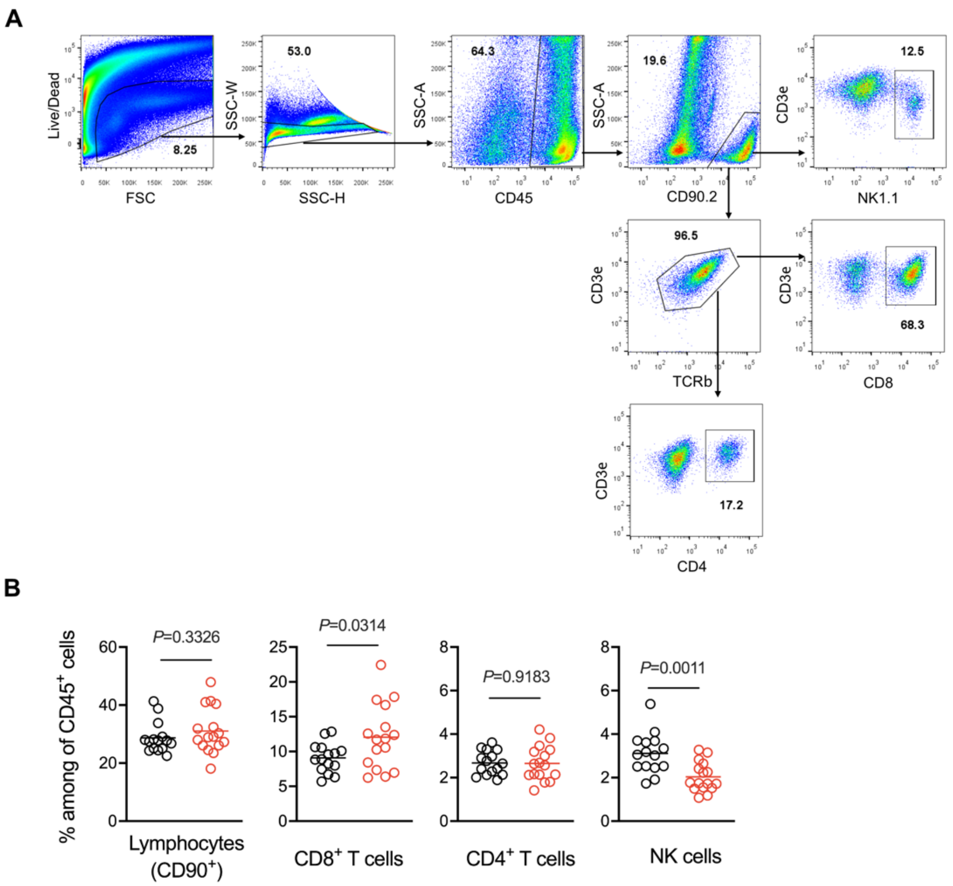

Figure S2. Analysis of tumor-infiltrating lymphoid cells in B16F10-dCas9 tumors.

(A) Flow cytometry gating strategy for lymphocytes in B16F10-dCas9 tumors.

(B) Percentages of tumor-infiltrating immune cell subsets among $CD45^+$ cells analyzed by flow cytometry comparing B16F10-dCas9 tumors expressing NTC sgRNA (n=15) versus *Mc1r* sgRNA (n=16). Lines indicate the mean. Welch's t-tests (two tailed) were used to determine statistical significance. Source data are provided as a Source Data file.

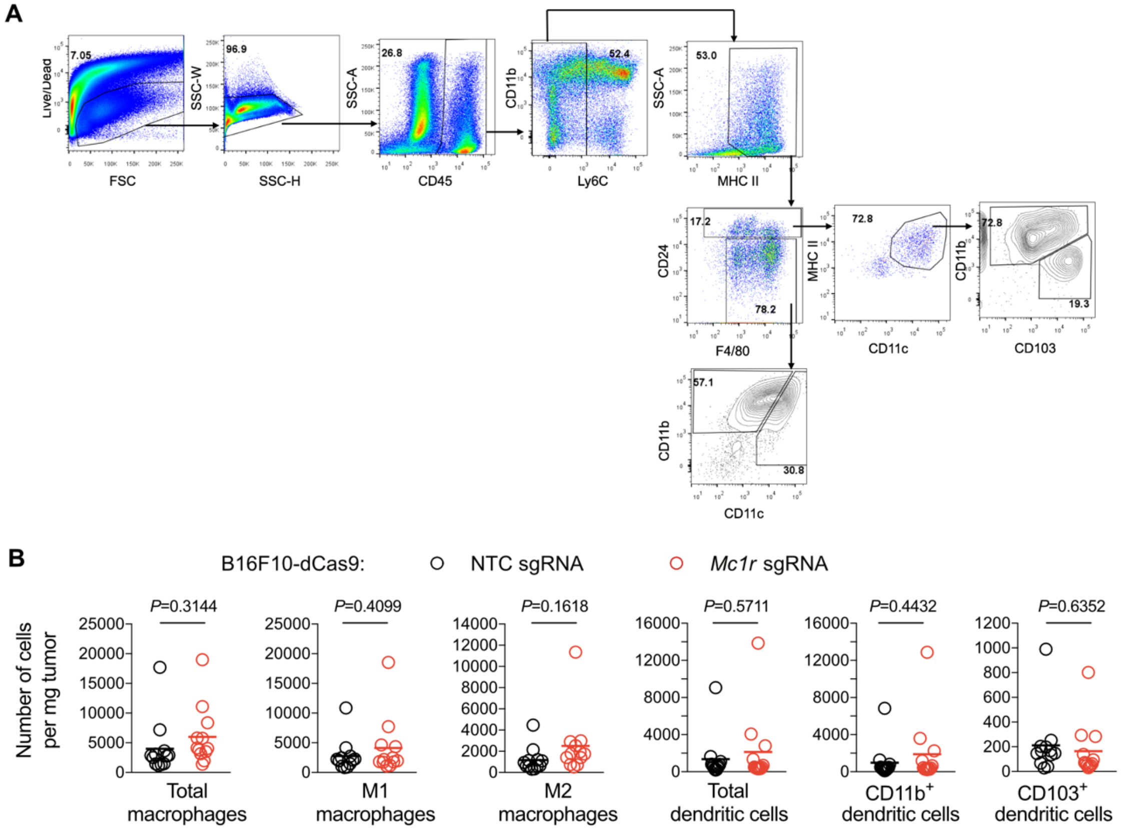

Figure S3. Analysis of tumor-infiltrating myeloid cells in B16F10-dCas9 tumors.

(A) Representative plots showing the gating strategy for myeloid cells in B16F10-dCas9 tumors.

(B) Number of tumor-infiltrating myeloid cell subsets per mg tumor analyzed by flow cytometry comparing B16F10-dCas9 tumors expressing NTC sgRNA (n=12) versus *Mc1r* sgRNA (n=12). Lines indicate the mean. Welch's t-tests (two tailed) were used to determine statistical significance. Source data are provided as a Source Data file.

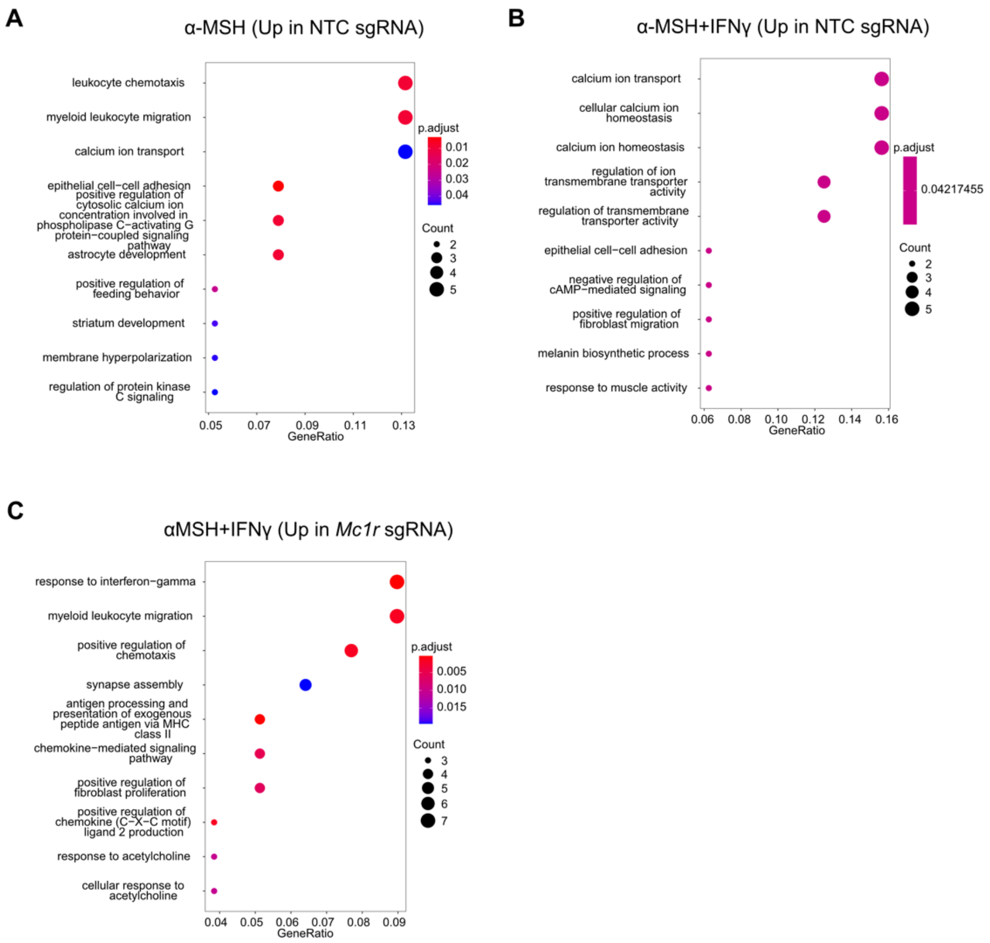

Figure S4. MC1R activation represses a subset of IFNγ-induced genes in B16F10 cells.

(A-C) Enriched gene ontology terms of differentially expressed genes indicated on Figure 3A.

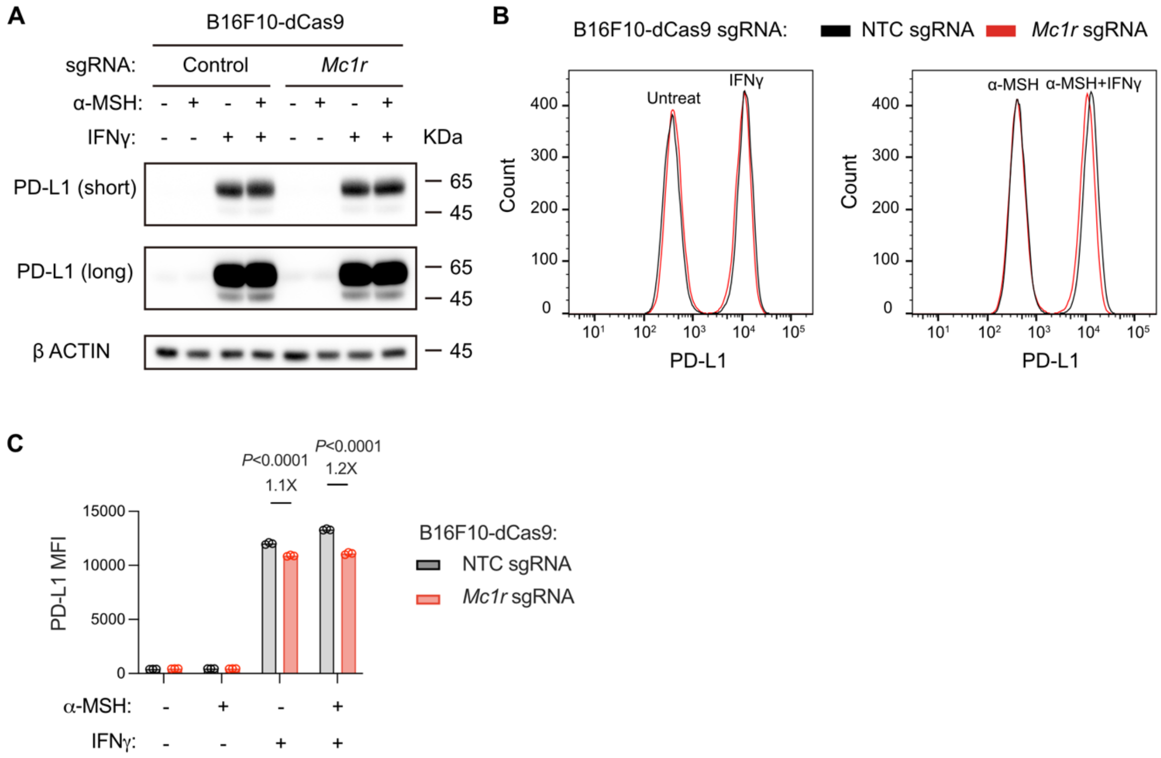

Figure S5. Impact of MC1R depletion on PD-L1 levels.

(A) Western blotting of the indicated proteins in B16F10-dCas9 cells transduced with indicated sgRNAs. Cells were untreated or treated with 1 μM α-MSH and 10 ng/ml IFNγ for 24 hours. A representative result was shown from two independent experiments. Uncropped western blot images are provided in Figure S11.

(B) Flow cytometry analysis of cell surface PD-L1 of B16F10-dCas9 cells transduced with indicated sgRNAs. Cells were untreated or treated with 1 μM α-MSH and 10 ng/ml IFNγ for 24 hours. A representative result was shown for three independent samples.

(C) MFI quantification of data (n=3 independent samples) in (B). Source data are provided as a Source Data file.

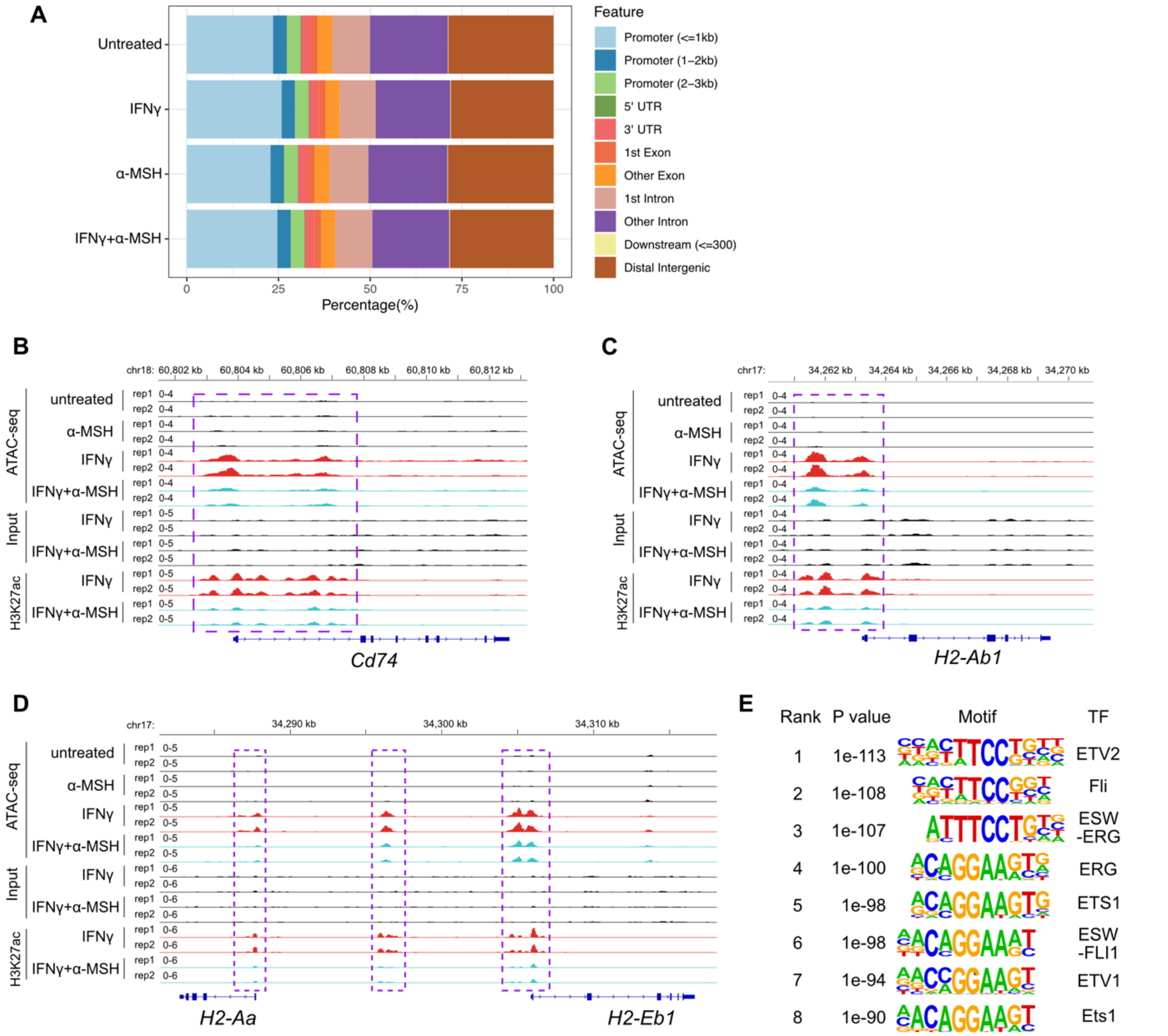

Figure S6. Impact of MC1R activation on chromatin accessibility.

(A) Genomic annotations of indicated classes of ATAC-seq peaks. Two independent samples were analyzed per treatment group.

(B-D) ATAC-seq and H3K27ac ChIP-seq tracks showing changes in chromatin accessibility and H3K27 acetylation at indicated genomic loci in B16F10 cells with indicated treatment of α-MSH (1 μM) and IFNγ (10 ng/ml) for 12 hours. Two independent samples were analyzed per treatment group.

(E) Top enriched motifs in chromatin sites with reduced accessibility in IFNγ+α-MSH treated cells relative to IFNγ treated cells. Two independent samples were analyzed per treatment group.

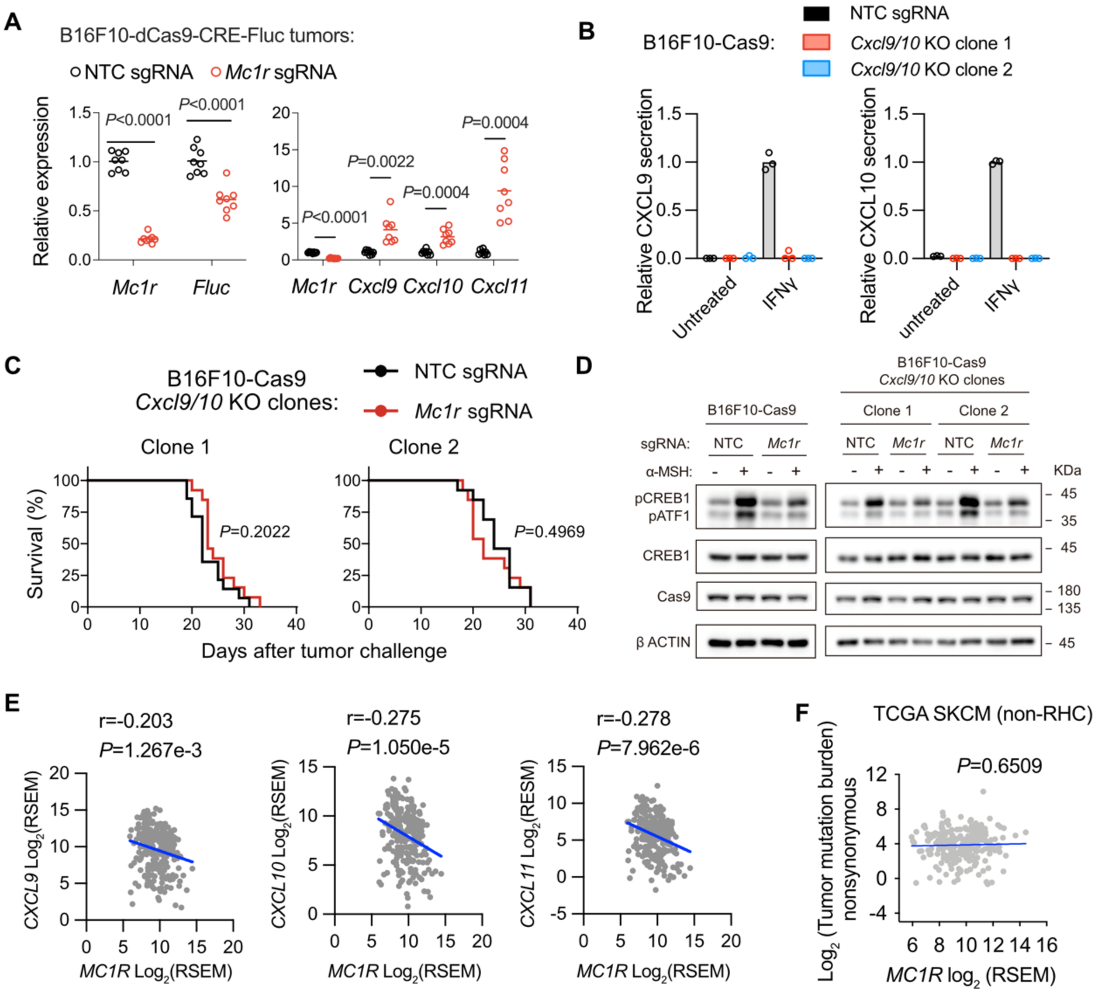

Figure S7. MC1R activation promotes immune evasion by repressing IFNγ-induced *Cxcl9/10* expression in B16F10 melanoma.

(A) qPCR quantification of *Mc1r*, *Fluc*, and *Cxcl9/10/11* mRNA levels in B16F10-dCas9-CRE-Fluc tumors transduced with NTC sgRNA or *Mc1r* sgRNA. Lines indicate the mean of eight independent tumors. Welch's t-tests (two-tailed) were used to determine statistical significance. Source data are provided as a Source Data file.

(B) Verification of two *Cxcl9/10* knockout clones by CXCL9 and CXCL10 ELISA. Cells were treated with IFNγ (10 ng/ml) for 24 hours. Three independent samples were analyzed per group. Source data are provided as a Source Data file.

(C) Survival analysis for the data shown in Figure 4E (B16F10-Cas9: n=8 for NTC sgRNA, 10 for *Mc1r* sgRNA; *Cxcl9/10* KO clone 1: n=14 for NTC sgRNA, 13 for *Mc1r* sgRNA; *Cxcl9/10* KO

clone 2: n=13 for NTC sgRNA, 13 for *Mc1r* sgRNA). The log-rank (Mantel-Cox) test was used to determine statistical significance. Source data are provided as a Source Data file.

(D) Western blotting of indicated proteins in B16F10-Cas9 cells or B16F10-Cas9 *Cxcl9*/*10* knockout cells transduced with NTC sgRNA or *Mc1r* sgRNA. Cells were treated with 1 μM α-MSH for 1 hour. A representative result was shown from two independent experiments. Uncropped western blot images are provided in Figure S11.

(E) Scatterplots displaying the correlation of *CXCL9*, *CXCL10*, and *CXCL11* expression levels with *MC1R* expression levels in non-RHC melanomas (n=250) from TCGA. Pearson correlation coefficients and two-tailed *P* values were indicated. RSEM: RNA-Seq by Expectation-Maximization. Source data are provided as a Source Data file.

(F) Scatterplot displaying the correlation of tumor mutation burden with *MC1R* expression levels in non-RHC melanomas (n=250) from TCGA. Pearson correlation coefficients and two-tailed *P* values were indicated. Source data are provided as a Source Data file.

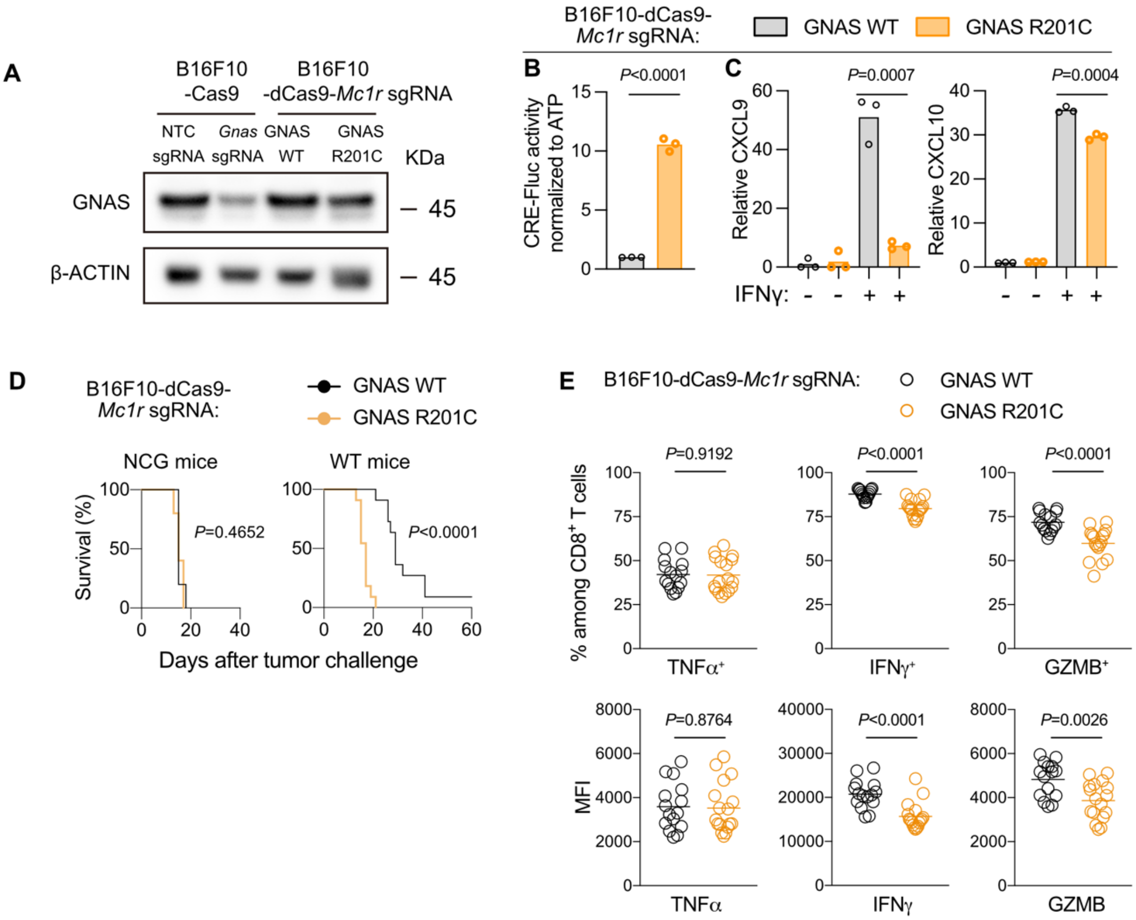

Figure S8. GNAS R201C promotes immune evasion and represses T cell infiltration in B16F10 melanoma.

(A) Western blotting of the indicated proteins in B16F10-dCas9 cells transduced with *Mc1r* sgRNA and indicated GNAS cDNAs. A representative result was shown from two independent experiments. Uncropped western blot images are provided in Figure S11.

(B) CRE-Fluc reporter activity of B16F10-dCas9-*Mc1r* sgRNA cells expressing wild-type GNAS or GNAS R201C mutant. Cells were treated with 1 μM α-MSH for 24 hours before measurements of CRE-Fluc reporter activity. Lines indicate the mean of three independent samples. The Student's *t*-test (two tailed) was used to determine statistical significance. Source data are provided as a Source Data file.

(C) ELISA quantification of CXCL9 and CXCL10 secretion by B16F10-dCas9-*Mc1r* sgRNA cells expressing wild-type GNAS or GNAS R201C mutant with indicated treatment of α-MSH (1 μM) and IFNγ (10 ng/ml) for 24 hours. Lines indicate the mean of three independent samples. Student's

t-tests (two-tailed) were used to determine statistical significance. Source data are provided as a Source Data file.

(D) Survival analysis for the data shown in Figure 6B (NCG mice: n=10 per group; WT mice: n=11 per group). Log-rank (Mantel-Cox) tests were used to determine statistical significance. Source data are provided as a Source Data file.

(E) Intracellular staining of TNFα, IFNγ, and GZMB of $CD8^+$ T cells from tumors in Figure 6C (n=15 for wild-type GNAS; n=17 for GNAS R201C mutant) after re-stimulation with Phorbol 12-myristate 13-acetate (PMA) and Ionomycin. Welch's t-test (two tailed) was used to determine statistical significance. Source data are provided as a Source Data file.

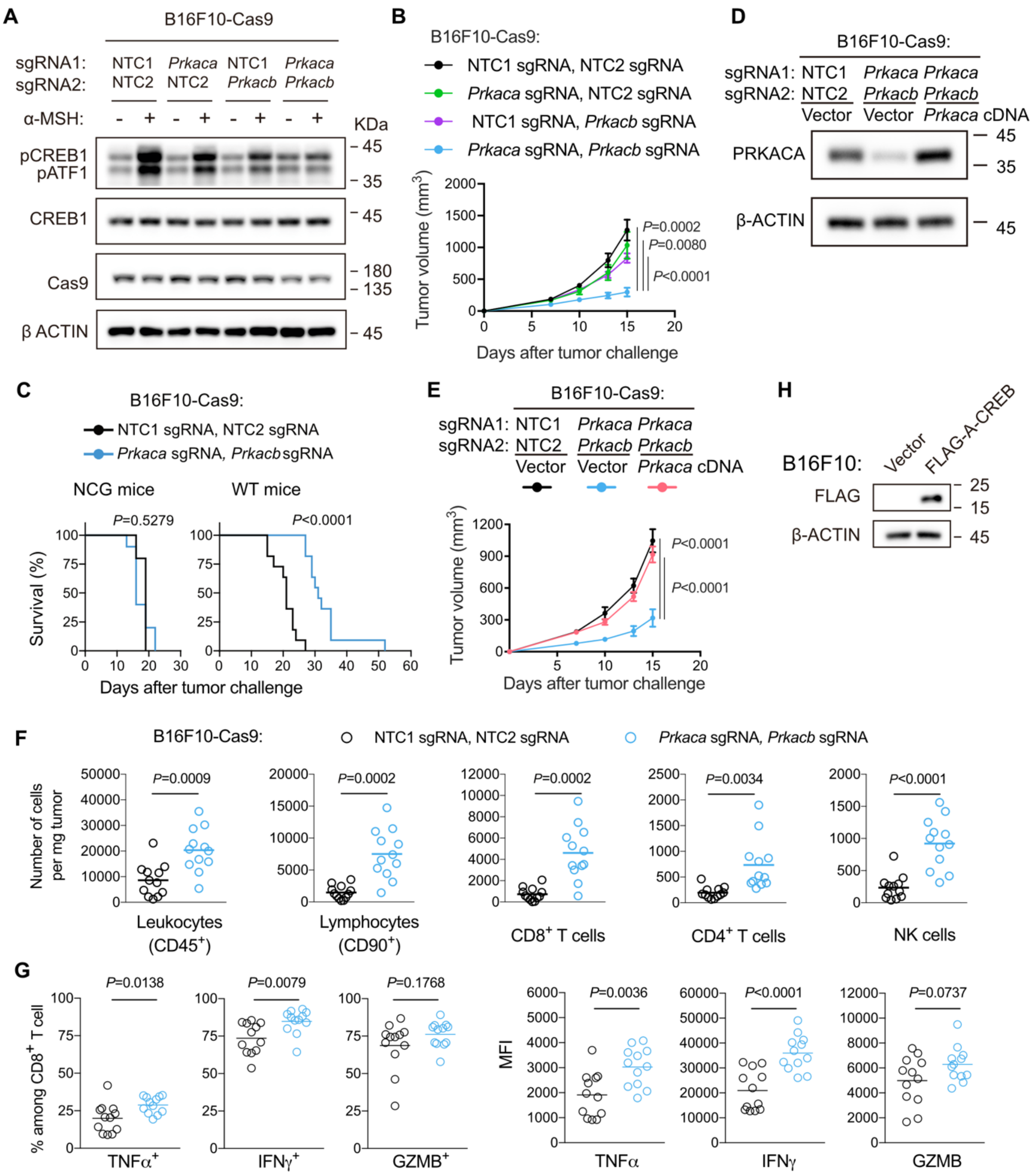

Figure S9. Inactivation of PKA activates T cell response in B16F10 melanoma.

(A) Western blotting of indicated proteins in B16F10-Cas9 cells transduced with combinations of sgRNAs targeting PKA paralogs. Cells were treated with 1 µM α-MSH for 1 hour. A representative result was shown from two independent experiments. Uncropped western blot images are provided in Figure S11.

(B) Tumor volume of wild-type mice transplanted with B16F10-Cas9 tumors expressing indicated combinations of sgRNAs. Data are the mean ± s.e.m. with n=9 animals per group (except for the NTC1 sgRNA, *Prkacb* sgRNA group, n=10). Welch's t-tests (two-tailed) were used to determine the statistical significance of the differences in tumor volume. Source data are provided as a Source Data file.

(C) Survival analysis for the data shown in Figure 6E (NCG mice: n=10 per group; WT mice: n=11 per group). The log-rank (Mantel-Cox) test was used to determine statistical significance. Source data are provided as a Source Data file.

(D) Western blotting of indicated proteins in B16F10-Cas9 cells transduced with indicated combinations of sgRNAs and cDNA. A representative result was shown from two independent experiments. Uncropped western blot images are provided in Figure S11.

(E) Tumor volume of wild-type mice transplanted with B16F10-Cas9 tumors expressing indicated combinations of sgRNAs and cDNA. Data are the mean ± s.e.m. with n=9 animals per group. Welch's t-tests (two-tailed) were used to determine the statistical significance of the differences in tumor volume. Source data are provided as a Source Data file.

(F) Number of tumor-infiltrating immune cell subsets per mg tumor analyzed by flow cytometry comparing B16F10-Cas9 tumors expressing NTC1/NTC2 sgRNAs (n=12) versus *Prkaca*/*Prkacb* sgRNAs (n=12). Lines indicate the mean. Welch's t-tests (two tailed) were used to determine statistical significance. Source data are provided as a Source Data file.

(G) Intracellular staining of TNFα, IFNγ, and GZMB of $CD8^+$ T cells from indicated tumors in (F) after re-stimulation. Welch's t-test (two tailed) was used to determine statistical significance. Source data are provided as a Source Data file.

(H) Detection of A-CREB expression by western blotting. A representative result was shown from two independent experiments. Uncropped western blot images are provided in Figure S11.

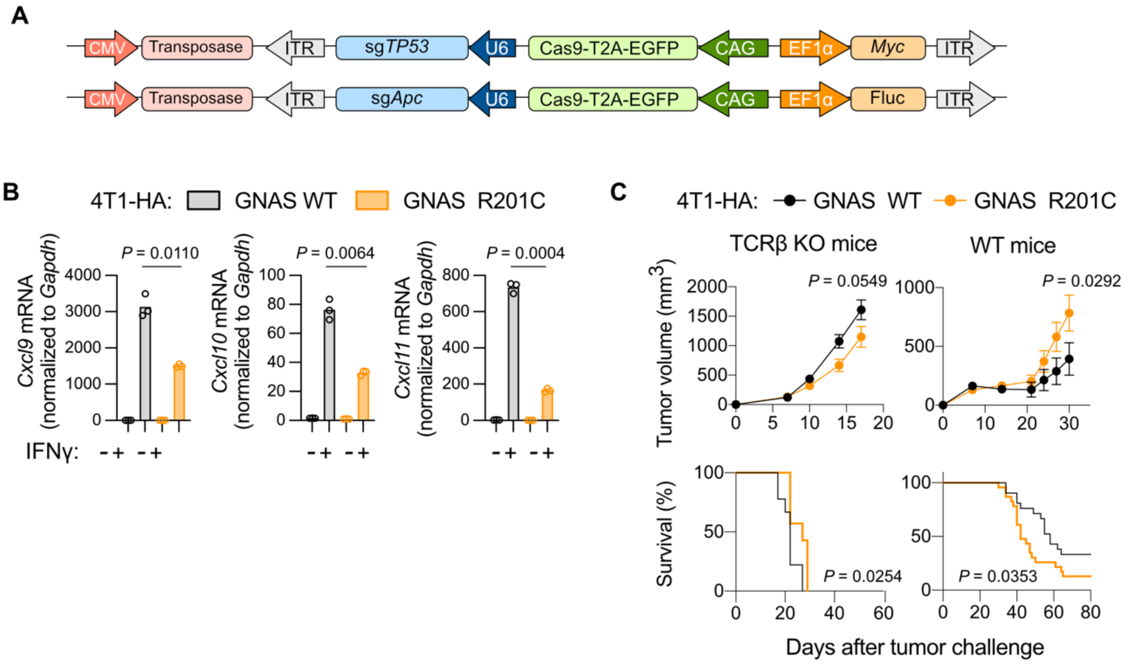

Figure S10. GNAS R201C promotes immune evasion in MAP and 4T1-HA tumors.

(A) Plasmid constructs used to induce liver cancer via hydrodynamic delivery.

(B) qPCR quantification of *Cxcl9/10/11* mRNA levels in 4T1-HA cells expressing wild-type GNAS or GNAS R201C mutant. Cells were treated with IFNγ (10 ng/ml) for 12 hours. Lines indicate the mean of three independent samples. Welch's t-tests (two-tailed) were used to determine statistical significance. Source data are provided as a Source Data file.

(C) Tumor volume and survival analysis of mice transplanted with 4T1-HA tumors expressing wild-type GNAS or GNAS R201C mutant in TCRβ KO mice (n=9 and 7 animals, respectively) and wild-type C57/BL6 mice (n=21 and 23, respectively). The Mann-Whitney test (two-tailed) and log-rank (Mantel-Cox) test were used to determine statistical significance in tumor volume and animal survival, respectively. Source data are provided as a Source Data file.

Figure S11. Uncropped wester blot images for supplementary figures

Figure S1 C

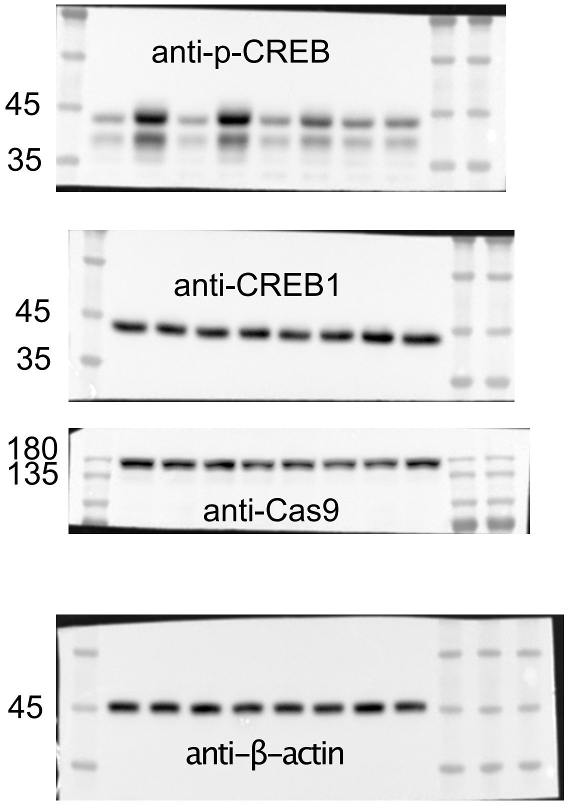

Figure S1 F

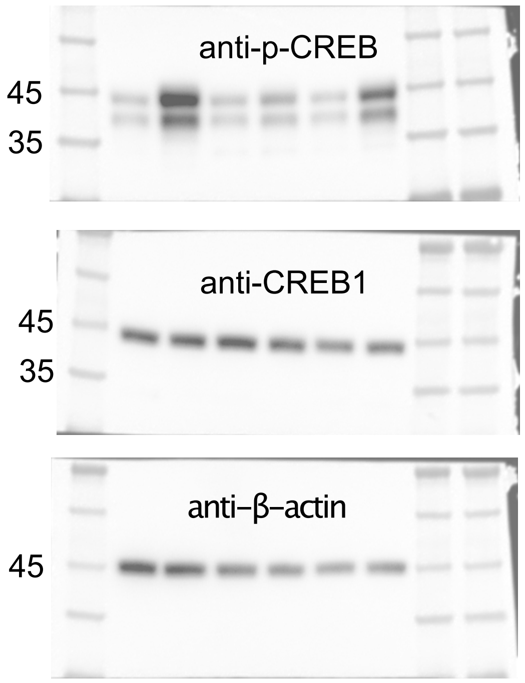

Figure S5 A

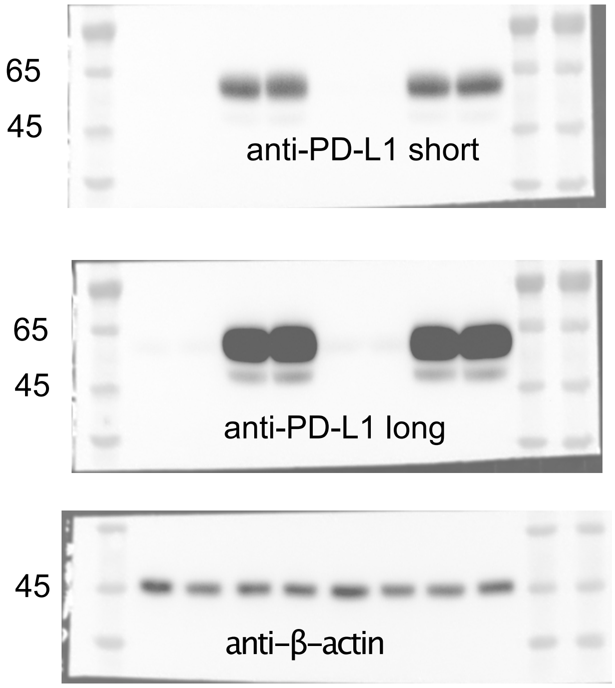

Figure S7D

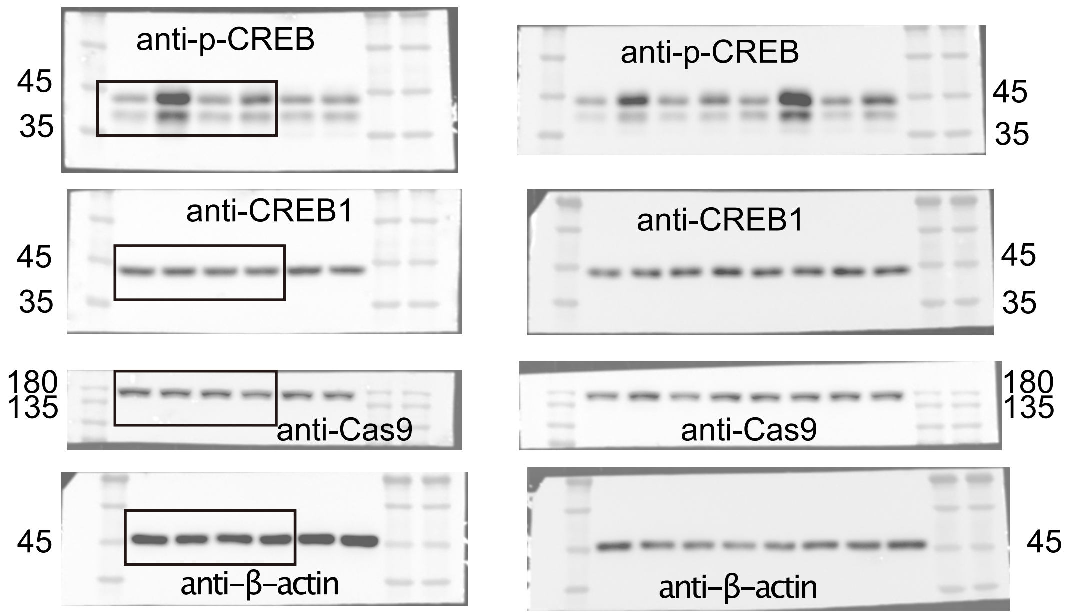

anti-p-CREB
45
35
anti-p-CREB
45
35
anti-CREB1
45
35
anti-CREB1
45
35
180
135
anti-Cas9
180
135
anti-Cas9
45
anti-β-actin
anti-β-actin
45

Figure S8A

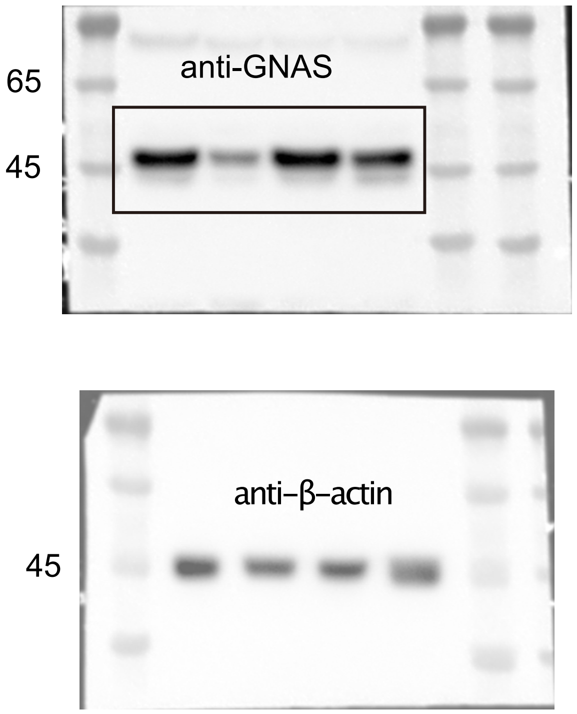

Figure S9A

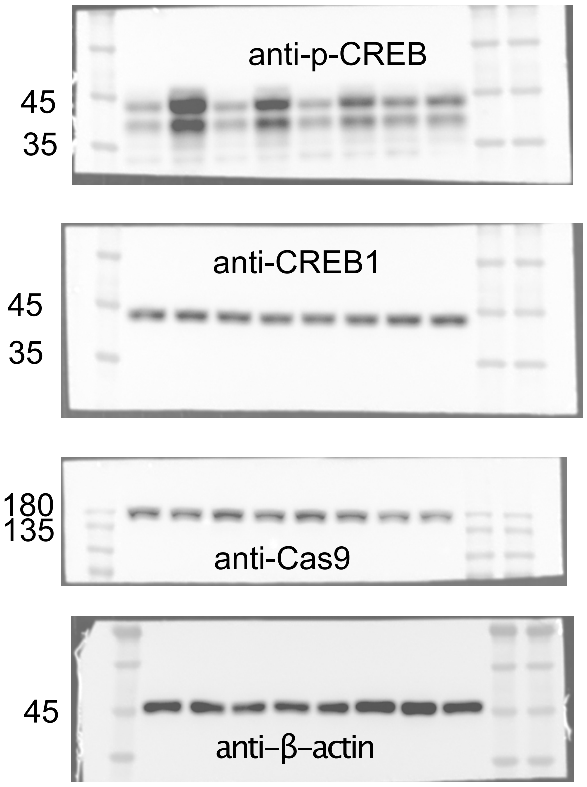

Figure S9D

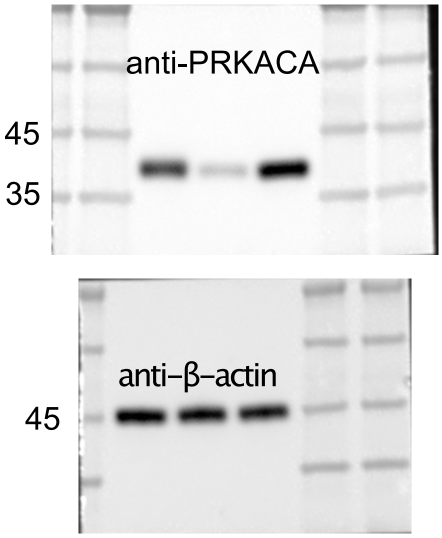

Figure S9H

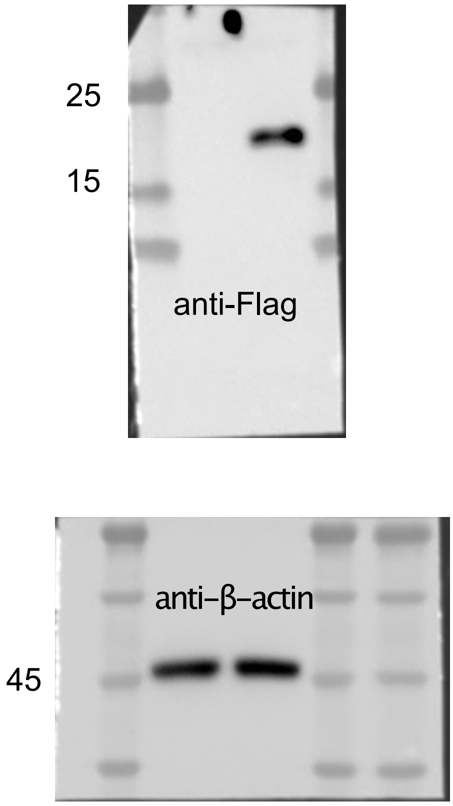

Supplement: Supplementary file 1 — Supplementary Information [file 41467_2023_41101_MOESM1_ESM.pdf]
