# Supplementary material for: Activation of melanocortin-1 receptor signaling in melanoma cells impairs T cell infiltration to dampen antitumor immunity
Source: Nat Commun. 2023 Sep 15;14:5740. doi: 10.1038/s41467-023-41101-3 (PMC10504282; doi:10.1038/s41467-023-41101-3)
Supplement: Supplementary file 3 — Description of Additional Supplementary Files [file 41467_2023_41101_MOESM3_ESM.pdf]

Description of Additional Supplementary Files

Title: Supplementary Data 1

Description: Raw sgRNA counts from indicated samples.
