# Supplementary material for: Activation of melanocortin-1 receptor signaling in melanoma cells impairs T cell infiltration to dampen antitumor immunity
Source: Nat Commun. 2023 Sep 15;14:5740. doi: 10.1038/s41467-023-41101-3 (PMC10504282; doi:10.1038/s41467-023-41101-3)
Supplement: Supplementary file 5 — Reporting Summary [file 41467_2023_41101_MOESM5_ESM.pdf]

nature portfolio

Corresponding author(s): Ting Han

Last updated by author(s): Aug 9, 2023

Reporting Summary

Nature Portfolio wishes to improve the reproducibility of the work that we publish. This form provides structure for consistency and transparency in reporting. For further information on Nature Portfolio policies, see our Editorial Policies and the Editorial Policy Checklist.

Statistics

For all statistical analyses, confirm that the following items are present in the figure legend, table legend, main text, or Methods section.

n/a	Confirmed	
☐	☒	The exact sample size (n) for each experimental group/condition, given as a discrete number and unit of measurement
☐	☒	A statement on whether measurements were taken from distinct samples or whether the same sample was measured repeatedly
☐	☒	The statistical test(s) used AND whether they are one- or two-sided<br>*Only common tests should be described solely by name; describe more complex techniques in the Methods section.*
☒	☐	A description of all covariates tested
☐	☒	A description of any assumptions or corrections, such as tests of normality and adjustment for multiple comparisons
☐	☒	A full description of the statistical parameters including central tendency (e.g. means) or other basic estimates (e.g. regression coefficient) AND variation (e.g. standard deviation) or associated estimates of uncertainty (e.g. confidence intervals)
☐	☒	For null hypothesis testing, the test statistic (e.g. F, t, r) with confidence intervals, effect sizes, degrees of freedom and P value noted<br>*Give P values as exact values whenever suitable.*
☒	☐	For Bayesian analysis, information on the choice of priors and Markov chain Monte Carlo settings
☒	☐	For hierarchical and complex designs, identification of the appropriate level for tests and full reporting of outcomes
☐	☒	Estimates of effect sizes (e.g. Cohen's d, Pearson's r), indicating how they were calculated

Our web collection on statistics for biologists contains articles on many of the points above.

Software and code

Policy information about availability of computer code

Data collection	Western blot images were collected using VILBER FUSION FX7 imager.<br>Flow cytometry data were collected using BD LSR Fortessa.<br>Immunofluorescence data were imaged using Zeiss LSM800 confocal microscope.
Data analysis	GraphPad Prism (v9.4.1), FlowJo (v10), R (v4.1), R Studio (v1.4.1106), MAGeCK (v0.5.9.2), Bowtie2 (v2.3.5.1), RSEM (v1.3.3), DESeq2 (v1.32.0), clusterProfiler (v4.0.5), fastp (v0.23.2), MACS2 (v2.2.7.1), Samtools (v0.1.1), deepTools (v3.4.3), IGV (v2.12.3), ChIPseeker (v1.28.3), and HOMER (v4.11).

For manuscripts utilizing custom algorithms or software that are central to the research but not yet described in published literature, software must be made available to editors and reviewers. We strongly encourage code deposition in a community repository (e.g. GitHub). See the Nature Portfolio guidelines for submitting code & software for further information.

Data

Policy information about availability of data

All manuscripts must include a data availability statement. This statement should provide the following information, where applicable:

- Accession codes, unique identifiers, or web links for publicly available datasets
- A description of any restrictions on data availability
- For clinical datasets or third party data, please ensure that the statement adheres to our policy

The RNA-seq, ATAC-seq, and ChIP-seq data generated in this study have been deposited in NCBI GEO (accession: GSE214859 [https://www.ncbi.nlm.nih.gov/geo/query/acc.cgi?acc=GSE214859] and GSE225553 [https://www.ncbi.nlm.nih.gov/geo/query/acc.cgi?acc=GSE225553]). The TCGA publicly available data used in this study are available in the cBioPortal database (https://www.cbioportal.org). The remaining data are available within the Article, Supplementary Information or Source Data file.

Human research participants

Policy information about studies involving human research participants and Sex and Gender in Research.

Reporting on sex and gender	none.
Population characteristics	none.
Recruitment	none.
Ethics oversight	none.

Note that full information on the approval of the study protocol must also be provided in the manuscript.

Field-specific reporting

Please select the one below that is the best fit for your research. If you are not sure, read the appropriate sections before making your selection.

[x] Life sciences [ ] Behavioural & social sciences [ ] Ecological, evolutionary & environmental sciences

For a reference copy of the document with all sections, see nature.com/documents/nr-reporting-summary-flat.pdf

Life sciences study design

All studies must disclose on these points even when the disclosure is negative.

Sample size	Sample size was empirically determined based on the intragroup variations of tumor growth and also based on previous publications (Manguso et al. Nature 2017; Ishizuka et al. Nature 2019).
Data exclusions	No data were excluded from the experiments.
Replication	Biological replicates were used in all experiments as noted in figure legends.
Randomization	Mice (age and sex-matched) were randomly divided into experimental groups.
Blinding	No blinding was used throughout experiments. The Investigators were not blinded to allocation during experiments and outcome assessment, because the data analyses were based on objectively measurable data.

Reporting for specific materials, systems and methods

We require information from authors about some types of materials, experimental systems and methods used in many studies. Here, indicate whether each material, system or method listed is relevant to your study. If you are not sure if a list item applies to your research, read the appropriate section before selecting a response.

Materials & experimental systems

n/a	Involved in the study
	☒ Antibodies
	☒ Eukaryotic cell lines
☒	Palaeontology and archaeology
	☒ Animals and other organisms
☒	Clinical data
☒	Dual use research of concern

Methods

n/a	Involved in the study
	☒ ChIP-seq
	☒ Flow cytometry
☒	MRI-based neuroimaging

Antibodies

Antibodies used

For western blotting: primary antibodies: anti-CREB (CST, 9197, dilution: 1:2000), anti-p-CREB (CST, 9198, dilution: 1:2000), anti-Cas9 (CST, 14697, dilution: 1:2000), anti-β-actin-HRP (Huaxingbio, HX18271, dilution: 1:10000), anti-GNAS (#10150-2-AP; Proteintech, Rosemont, USA; dilution:1:2000), anti-PD-L1 (#ab213480; Abcam, Waltham, USA; dilution:1:2000) . Secondary antibodies: Anti-rabbit IgG, HRP-linked Antibody (CST, 7074, dilution: 1:10000), Peroxidase-Conjugated Goat anti-Mouse IgG (ZSGB-Bio, ZB-2305, dilution: 1:10000).
For flow cytometry: Anti-Mouse CD45-APC/Cyanine7 (Biolegend, 103116, clone: 30-F11, dilution:1:400), Anti-Mouse CD90.2-PerCP/Cyanine5.5 (Biolegend, 105338, clone: 30-H12, dilution:1:400), Anti-Mouse CD4-BV421 (Biolegend, 100438, clone: GK1.5, dilution:1:400), Anti-Mouse TCR β-Alexa Fluor® 700 (Biolegend, 109224, clone: H57-597, dilution:1:400), Anti-Mouse CD3ε-PE/Cyanine7 (Biolegend, 100320, clone: 145-2C11, dilution:1:400), Anti-Mouse CD8a-BUV395 (BD Biosciences, 563786, clone: 53-6.7, dilution:1:400), Anti-Mouse TCR β-APC/Cyanine7 (Biolegend, 109220, clone: H57-597, dilution:1:400), Anti-Mouse CD3-Alexa Fluor® 700 (Biolegend, 100216, clone: 17A2, dilution:1:400), Anti-human/mouse Granzyme B-FITC (Biolegend, 515403, clone: GB11, dilution:1:400), Anti-mouse TNFα BV650 (Biolegend, 506333, clone: MP6-XT22, dilution:1:400), Anti-Mouse IFNγ-PE/Cyanine7 (Biolegend, 505826, clone: XMG1.2, dilution:1:400), Anti-mouse CD16/CD32 (BioXcell, BE0307, clone: 2.4G2, dilution:1:400), Anti-Mouse CD24-BV421 (Biolegend, 101826, clone: M1/69, dilution:1:400), Anti-Mouse CD45-BV650 (Biolegend, 103151, clone: 30-F11, dilution:1:400), Anti-Mouse CD11c-PE/Cyanine7 (Biolegend, 117318, clone: N418, dilution:1:400), Anti-Mouse CD11b-APC-eFluor 780 (eBioscience, 47-0112-82, clone: M1/70, dilution:1:400), Anti-Mouse Ly-6C-PerCP/Cyanine5.5 (Biolegend, 128012, clone: HK1.4, dilution:1:400), Anti-Mouse I-A/I-E-Alexa Fluor® 700 (Biolegend, 107622, clone: M5/114.15.2, dilution:1:400), Anti-Mouse CD103-PE (Biolegend, 121406, clone: 2E7, dilution:1:400), Anti-Mouse F4/80-Biotin (Biolegend, 123106, clone: BM8, dilution:1:400), BUV395 Streptavidin (BD Biosciences, 564176, dilution:1:1000), anti-mouse PD-L1 (Biolegend, 124311, clone:10F.9G2, dilution:1:400) .
For immunofluorescence: Anti-CD8α (Abcam, ab217344, clone: EPR21769, dilution:1:500), donkey anti-rabbit Alexa fluor647 (Jackson Immuno Research, 711-605-152, clone: 30-F11, dilution:1:500).
For ChIP seq: H3K27ac (Abcam, ab4729, 10 µg for 15 million cells).

Validation

All the commercial antibodies were validated by the suppliers.
anti-CREB: https://www.cellsignal.com/products/primary-antibodies/creb-48h2-rabbit-mab/9197
anti-p-CREB: https://www.cellsignal.com/products/primary-antibodies/phospho-creb-ser133-87g3-rabbit-mab/9198
anti-Cas9: https://www.cellsignal.com/products/primary-antibodies/cas9-s-pyogenes-7a9-3a3-mouse-mab/14697
anti-β-actin-HRP: http://www.huaxingbio.com/pd.jsp?id=63&nSL=%5B%2C6%2C7%5D#skeyword=HX18271&_pp=0_35
anti-GNAS: https://www.ptgcn.com/products/NESP55,GNAS-Antibody-10150-2-AP.htm
anti-PD-L1: https://www.abcam.com/products/primary-antibodies/pd-l1-antibody-epr20529-ab213480.html
Anti-rabbit IgG, HRP-linked Antibody: https://www.cellsignal.com/products/secondary-antibodies/anti-rabbit-igg-hrp-linked-antibody/7074?bvstate=pg:2/ct:r
Peroxidase-Conjugated Goat anti-Mouse IgG: http://www.zsbio.com/product/ZB-2305
Anti-Mouse CD45-APC/Cyanine7: https://www.biolegend.com/de-at/products/apc-cyanine7-anti-mouse-cd45-antibody-2530
Anti-Mouse CD90.2-PerCP/Cyanine5.5: https://www.biolegend.com/de-at/products/percp-cyanine5-5-anti-mouse-cd90-2-thy1-2-antibody-12938
Anti-Mouse CD4-BV421: https://www.biolegend.com/de-at/products/brilliant-violet-421-anti-mouse-cd4-antibody-7142
Anti-Mouse TCR β-Alexa Fluor® 700: https://www.biolegend.com/de-at/products/alexa-fluor-700-anti-mouse-tcr-beta-chain-antibody-4537
Anti-Mouse CD3ε-PE/Cyanine7: https://www.biolegend.com/de-at/products/pe-cyanine7-anti-mouse-cd3epsilon-antibody-1899
Anti-Mouse CD8a-BUV395: https://www.bdbiosciences.com/en-us/products/reagents/flow-cytometry-reagents/research-reagents/single-color-antibodies-ruo/buv395-rat-anti-mouse-cd8a.563786
Anti-Mouse TCR β-APC/Cyanine7: https://www.biolegend.com/de-at/products/apc-cyanine7-anti-mouse-tcr-beta-chain-antibody-4137
Anti-Mouse CD3-Alexa Fluor® 700: https://www.biolegend.com/de-at/products/alexa-fluor-700-anti-mouse-cd3-antibody-3375
Anti-human/mouse Granzyme B-FITC: https://www.biolegend.com/de-at/products/fitc-anti-human-mouse-granzyme-b-antibody-6066
Anti-mouse TNFα BV650: https://www.biolegend.com/de-at/products/brilliant-violet-650-anti-mouse-tnf-alpha-antibody-8829
Anti-Mouse IFNγ-PE/Cyanine7: https://www.biolegend.com/de-at/products/pe-cyanine7-anti-mouse-ifn-gamma-antibody-5865
Anti-mouse CD16/CD32: https://bioxcell.com/invivomab-anti-mouse-cd16-cd32
Anti-Mouse CD24-BV421: https://www.biolegend.com/de-at/products/brilliant-violet-421-anti-mouse-cd24-antibody-7323
Anti-Mouse CD45-BV650: https://www.biolegend.com/de-at/products/brilliant-violet-650-anti-mouse-cd45-antibody-11987
Anti-Mouse CD11c-PE/Cyanine7: https://www.biolegend.com/de-at/products/pe-cyanine7-anti-mouse-cd11c-antibody-3086
Anti-Mouse CD11b-APC-eFluor 780: https://www.thermofisher.cn/cn/zh/antibody/product/CD11b-Antibody-clone-M1-70-Monoclonal/47-0112-82
Anti-Mouse Ly-6C-PerCP/Cyanine5.5: https://www.biolegend.com/de-at/products/percp-cyanine5-5-anti-mouse-ly-6c-antibody-5967

Anti-Mouse I-A/I-E-Alexa Fluor® 700: https://www.biolegend.com/de-at/products/alexa-fluor-700-anti-mouse-i-a-i-e-antibody-3413
Anti-Mouse CD103-PE: https://www.biolegend.com/de-at/products/pe-anti-mouse-cd103-antibody-3574
Anti-Mouse F4/80-Biotin: https://www.biolegend.com/de-at/products/biotin-anti-mouse-f4-80-antibody-4066
BUV395 Streptavidin: https://www.bdbiosciences.com/en-au/products/reagents/flow-cytometry-reagents/research-reagents/single-color-antibodies-ruo/buv395-streptavidin.564176
anti-mouse PD-L1: https://www.biolegend.com/de-at/products/apc-anti-mouse-cd274-b7-h1-pd-l1-antibody-6655
Anti-CD8α: https://www.abcam.com/products/primary-antibodies/cd8-alpha-antibody-epr21769-ab217344.html
donkey anti-rabbit Alexa fluor647: https://www.jacksonimmuno.com/catalog/products/711-605-152
H3K27ac: https://www.abcam.com/products/primary-antibodies/histone-h3-acetyl-k27-antibody-chip-grade-ab4729.html

Eukaryotic cell lines

Policy information about cell lines and Sex and Gender in Research

Cell line source(s)	B16F10, MCF-7, SiHa, A875, and A375 were purchased from Cell Resource Center, Peking Union Medical College (Beijing, China). HCmel1274 was purchased from the American Type Culture Collection (ATCC). LS513 was purchased from Meisen Cell, Zhejiang, China. SKMEL2, SKMEL28, SKMEL24, Hs695T, Malme-3M, C32, SW756, 786-O, and OSRC2 were purchased from Cobioer, Nanjing, China. 4T1 was a gift from Dr. Xiaodong Wang (NIBS, Beijing, China). TMD8 was a gift from BeiGene Co., Ltd., Beijing, China. HEK293T, HeLa, LoVo, SW48, NCI-H1581, NCI-H2030, and U2OS were gifts from Dr. Deepak Nijhawan (University of Texas Southwestern Medical Center, Dallas, Texas, USA). MAP was derived in house.
Authentication	None of the cell lines were independently authenticated by us.
Mycoplasma contamination	All cell lines were tested negative for mycoplasma contamination using routine PCR-based assay.
Commonly misidentified lines (See ICLAC register)	None.

Animals and other research organisms

Policy information about studies involving animals; ARRIVE guidelines recommended for reporting animal research, and Sex and Gender in Research

Laboratory animals	For in vivo tumor challenge experiments, all animals were 6–10-week-old female mice. Wild-type C57BL/6 mice and BALB/c mice were obtained from the Transgenic Research Center at NIBS or were purchased from Beijing Vital River Laboratory Animal Technology (Beijing, China). NCG (NOD/ShiLtJGpt-Prkdcem26Cd52Il2rgem26Cd22/Gpt) mice were purchased from GemPharmatech (Nanjing, Jiangsu, China). TCRβ knockout mice (JAX stock# 002118), Rag1 knockout mice (JAX stock#002216) and Cas9 transgenic mice (JAX stock# 026179) were bred in house.
Wild animals	This study did not involve wild animals.
Reporting on sex	All tumor challenge experiments were performed on female mice.
Field-collected samples	This study did not involve field-collected samples.
Ethics oversight	All mice were housed in the specific-pathogen free animal facility at NIBS under a 12-h light–dark cycle with free access to food and water, 23 °C–25 °C and 50%–56% humidity. All animal experiments were approved by the NIBS Animal Use and Care Committee.

Note that full information on the approval of the study protocol must also be provided in the manuscript.

ChIP-seq

Data deposition

☒ Confirm that both raw and final processed data have been deposited in a public database such as GEO.

☒ Confirm that you have deposited or provided access to graph files (e.g. BED files) for the called peaks.

Data access links *May remain private before publication.*	https://www.ncbi.nlm.nih.gov/geo/query/acc.cgi?acc=GSE225553. Taken code:crcjcgywllchxil
Files in database submission	GSM7050259 B16F10_ATACseq_untreat_rep1<br>GSM7050260 B16F10_ATACseq_untreat_rep2<br>GSM7050261 B16F10_ATACseq_aMSH_rep1<br>GSM7050262 B16F10_ATACseq_aMSH_rep2<br>GSM7050263 B16F10_ATACseq_IFNg_rep1<br>GSM7050264 B16F10_ATACseq_IFNg_rep2<br>GSM7050265 B16F10_ATACseq_aMSH+IFNg_rep1<br>GSM7050266 B16F10_ATACseq_aMSH+IFNg_rep2<br>GSM7050267 B16F10_H3K27ac_chipseq_IFNg_input_rep1<br>GSM7050268 B16F10_H3K27ac_chipseq_IFNg_input_rep2<br>GSM7050269 B16F10_H3K27ac_chipseq_aMSH+IFNg_input_rep1

GSM7050270 B16F10_H3K27ac_chipseq_aMSH+IFNg_input_rep2
GSM7050271 B16F10_H3K27ac_chipseq_IFNg_chip_rep1
GSM7050272 B16F10_H3K27ac_chipseq_IFNg_chip_rep2
GSM7050273 B16F10_H3K27ac_chipseq_aMSH+IFNg_chip_rep1
GSM7050274 B16F10_H3K27ac_chipseq_aMSH+IFNg_chip_rep2

Genome browser session (e.g. UCSC): IGV (2.15.2)

Methodology

Replicates: ATAC seq and H3K27ac ChIP seq have two replicates for each condition.

Sequencing depth:
B16F10_ATACseq_untreat_rep1: 300965 (16.23%) aligned exactly 1 time, 644627 (34.75%) aligned >1 times, 97.90% overall alignment rate, paired-end.
B16F10_ATACseq_untreat_rep2: 257184 (16.04%) aligned exactly 1 time, 564110 (35.17%) aligned >1 times, 98.07% overall alignment rate, paired-end.
B16F10_ATACseq_aMSH_rep1: 310019 (16.35%) aligned exactly 1 time, 733010 (38.66%) aligned >1 times, 97.98% overall alignment rate, paired-end.
B16F10_ATACseq_aMSH_rep2: 311954 (17.10%) aligned exactly 1 time, 734562 (40.27%) aligned >1 times, 98.00% overall alignment rate, paired-end.
B16F10_ATACseq_IFNg_rep1: 336121 (17.87%) aligned exactly 1 time, 760741 (40.44%) aligned >1 times, 98.01% overall alignment rate, paired-end.
B16F10_ATACseq_IFNg_rep2: 562267 (19.01%) aligned exactly 1 time, 1219395 (41.22%) aligned >1 times, 98.02% overall alignment rate, paired-end.
B16F10_ATACseq_aMSH+IFNg_rep1: 405818 (18.31%) aligned exactly 1 time, 872718 (39.39%) aligned >1 times, 97.81% overall alignment rate, paired-end.
B16F10_ATACseq_aMSH+IFNg_rep2: 239207 (14.86%) aligned exactly 1 time, 592276 (36.79%) aligned >1 times, 97.91% overall alignment rate, paired-end.
B16F10_H3K27ac_chipseq_IFNg_input_rep1: 1826771 (20.06%) aligned exactly 1 time, 3450373 (37.88%) aligned >1 times, 96.49% overall alignment rate, paired-end.
B16F10_H3K27ac_chipseq_IFNg_input_rep2: 1293396 (20.57%) aligned exactly 1 time, 2458879 (39.10%) aligned >1 times, 96.09% overall alignment rate, paired-end.
B16F10_H3K27ac_chipseq_aMSH+IFNg_input_rep1: 1596406 (19.21%) aligned exactly 1 time, 3177385 (38.23%) aligned >1 times, 96.28% overall alignment rate, paired-end.
B16F10_H3K27ac_chipseq_aMSH+IFNg_input_rep2: 1697763 (18.89%) aligned exactly 1 time, 3431452 (38.19%) aligned >1 times, 95.57% overall alignment rate, paired-end.
B16F10_H3K27ac_chipseq_IFNg_chip_rep1: 1354874 (30.02%) aligned exactly 1 time, 795512 (17.63%) aligned >1 times, 96.43% overall alignment rate, paired-end.
B16F10_H3K27ac_chipseq_IFNg_chip_rep2: 1528769 (29.30%) aligned exactly 1 time, 899943 (17.25%) aligned >1 times, 96.05% overall alignment rate, paired-end.
B16F10_H3K27ac_chipseq_aMSH+IFNg_chip_rep1: 1952434 (29.62%) aligned exactly 1 time, 1112638 (16.88%) aligned >1 times, 96.18% overall alignment rate, paired-end.
B16F10_H3K27ac_chipseq_aMSH+IFNg_chip_rep2: 1956274 (29.45%) aligned exactly 1 time, 1146764 (17.26%) aligned >1 times, 96.05% overall alignment rate, paired-end.

Antibodies: H3K27ac (ab4729).

Peak calling parameters: macs2 callpeak -g mm -f BAMPE --broad -t ${sample}.bam --bdg -n ${sample} --outdir peakcalling/${sample}

Data quality: Peaks are at FDR 5% and between 5-fold enrichment and 50-fold enrichment.

Software: Bowtie2 (2.3.5.1), Samtools (0.1.1), MACS2 (2.2.7.1), Deeptools (3.4.3), Chipseeker (1.28.3), Homer findMotifsGenome.pl (4.9.1).

Flow Cytometry

Plots

Confirm that:

- [x] The axis labels state the marker and fluorochrome used (e.g. CD4-FITC).
- [x] The axis scales are clearly visible. Include numbers along axes only for bottom left plot of group (a 'group' is an analysis of identical markers).
- [x] All plots are contour plots with outliers or pseudocolor plots.
- [x] A numerical value for number of cells or percentage (with statistics) is provided.

Methodology

Sample preparation: In total, 2 million tumor cells were subcutaneously injected into C57BL/6 mice. After 12-14 days, tumor tissues were dissected out and then minced and dissociated in RPMI-1640 containing collagenase (1 mg/ml collagenase I; Roche, Basel,

	Switzerland) and DNase I (200 µg/ml; Sigma, St. Louis, Missouri, USA) with constant stirring at 37 °C for 20 minutes. Single cells were obtained by filtering through a 100-micron filter.
Instrument	LSR Fortessa (BD Biosciences).
Software	FlowJo (v10).
Cell population abundance	Tumour-infiltrating immune cells were analysed based on immune cells makers by flow cytometry.
Gating strategy	All gates were set based on naive mice spleen control and isotype control antibodies after appropriate compensation using single-stained compensation controls.

☒ Tick this box to confirm that a figure exemplifying the gating strategy is provided in the Supplementary Information.
